# Transverse cerebellar diameter: a reliable predictor of gestational age

**DOI:** 10.4314/ahs.v20i4.51

**Published:** 2020-12

**Authors:** Sanjay Mishra, Surajit Ghatak, Pratibha Singh, Dushyant Agrawal, Pawan Garg

**Affiliations:** 1 All India Institute of Medical Sciences Jodphur, Department of Anatomy; 2 All India Institute of Medical Sciences Jodphur, Department of Obstetrics and Gynaecology; 3 All India Institute of Medical Sciences Jodphur, Department of Radiodiagnosis

**Keywords:** Transverse cerebellar diameter, ultrasonography, gestational age

## Abstract

**Objectives:**

To determine accuracy of transverse cerebellar diameter (TCD) measurement in the prediction of gestational age (GA) in normal fetuses; to develop reference chart for TCD according to GA in Indian population.

**Design:**

A retrospective cross-sectional study.

**Method:**

Ultrasonographic measurements in 300 singleton pregnant women included biparietal diameter (cm), head circumference (cm), abdominal circumference (cm), femur length (cm) and transverse cerebellar diameter (cm). Reference chart with mean TCD for corresponding gestational age (GA) in weeks was developed.

**Results:**

Statistically significant relationship found between TCD and gestational age (R2=0.92, p=0.0006). Regression formulae based on TCD with other parameter can be used to predict gestational age of foetus. When TCD is compared with findings in other studies in different ethnic population, it is found that there is significant difference exists.

**Conclusion:**

In normally developing fetuses the TCD has linear correlation with advancing gestational age. A separate reference chart is required for every different population because ethnicity, nutrition and environmental factors can have impact on normal TCD values. This will help to avoid misinterpretation of data to determine gestational age.

## Introduction

Correct assessment of gestational age (GA) and fetal growth is essential for optimal obstetric^1^ management at the time of delivery. The biometric parameters used most commonly to evaluate fetal growth include biparietal diameter (BPD), head circumference (HC), abdominal^2^ circumference (AC) and femur length (FL). These parameters have few limitations as condition altering the shape of skull like dolicocephaly and brachycephaly will affect the BPD^3^,^4^,^5^, which is well accepted indicator of GA. Femur length (FL) varies somewhat with ethnicity. Transverse cerebellar diameter measurement is emerging as new biometric parameter to assess fetal growth^6^,^7^,^8^,^9^. TCD is rarely affected in conditions altering shape of skull because it is 10 deeply located in brain and protected by bony petrous ridges.

The objective for this study was to assess reliability of TCD in predicting gestational age as an independent parameter. Most of the ultrasound machines are fed with data obtained from western population which can differ with other population due to ethnic, nutrition and environmental factor. A separate reference chart for Indian population is needed because there might be a risk of over-diagnosing intrauterine growth retardation.

## Materials and methods

Retrospective cross-sectional study was carried out at All India Institute of Medical Sciences, Jodhpur from September 2017 to April 2019. Prior permission from institutional ethical committee was taken for the same study. The study included 300 normal singleton pregnancies which were referred for routine fetal anomaly scan from 14- 40 weeks of gestation.

**Inclusion criteria:**

1. Maternal age from 18 to 45 years
2. 14 to 40 weeks of gestation
3. Regular menses with known LMP

**Exclusion criteria:** 1. Any congenital anomalies of fetus (Anencephaly, Chiari malformation, hydromnios, conjoined twins etc.)

**Measurement of various parameters:** All measurements were made by scanning the patients using LOGIQ S8 R2, WIPRO GE -400 PRO Version USG machine. In each patient BPD, HC, AC, FL and TCD were measured.

**Fig. 1:**
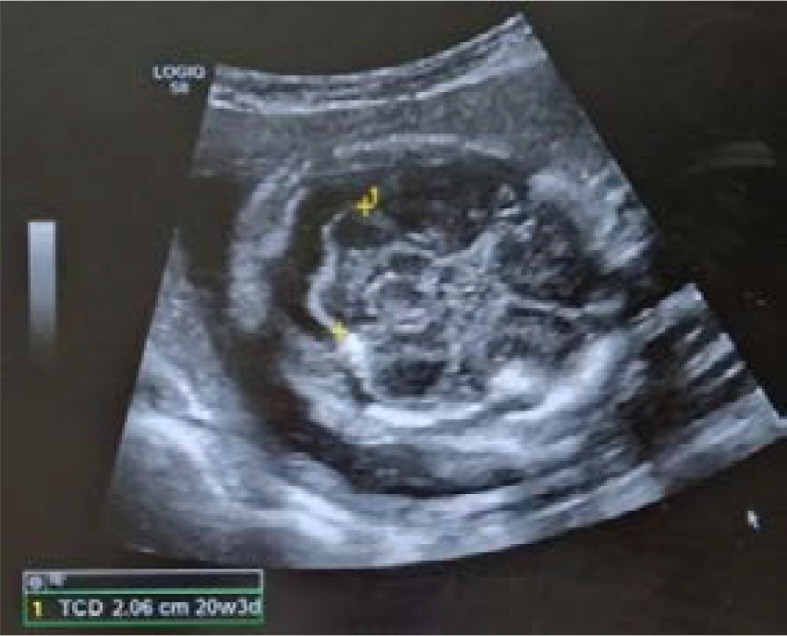
Showing measurement of TCD in USG machine.

**TCD:** The cerebellar view is obtained by rotating the transducer in the axial plane centered on the thalamus to show the cerebellar hemispheres. This view shows cerebellum, the cistern magna and the cavum septum pellucidi. The cerebellum characteristically appears as two lobules on either side of the midline in the posterior cranial fossa. The widest diameter of the cerebellum is measured (Fig. 1).

**Fig. 2:**
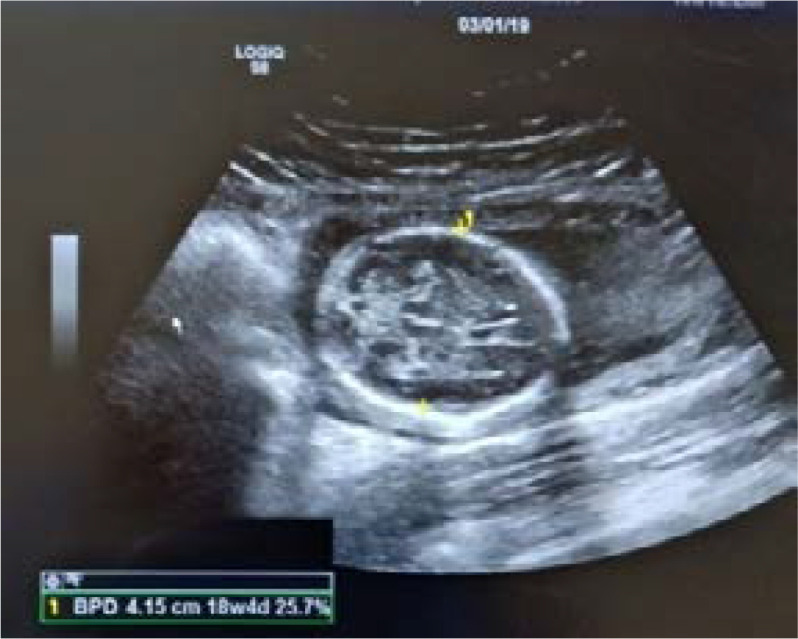
Showing measurement of BPD in USG machine.

**BPD:** Measurement was taken from trans-axial sonograms of fetal head at the level of paired thalami and cavum septum pellucidi. The BPD is measured from the outer edge of the cranium nearest the transducer to the inner edge of cranium farthest from the transducer (Fig. 2).

**Fig. 3:**
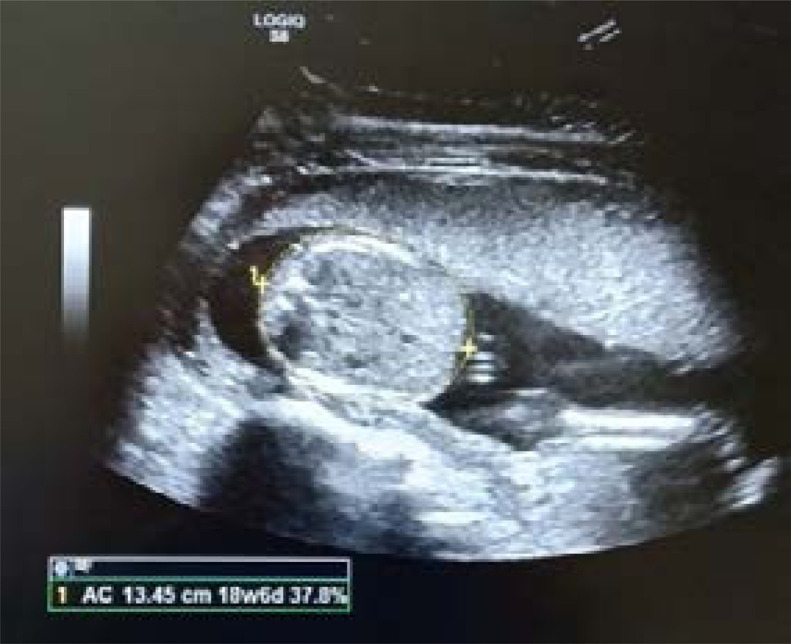
Showing measurement of AC in USG machine.

**AC:** Measurement was taken on transverse scan at the level of stomach and intrahepatic portion of the umbilical vein (Fig. 3).

**Fig. 4:**
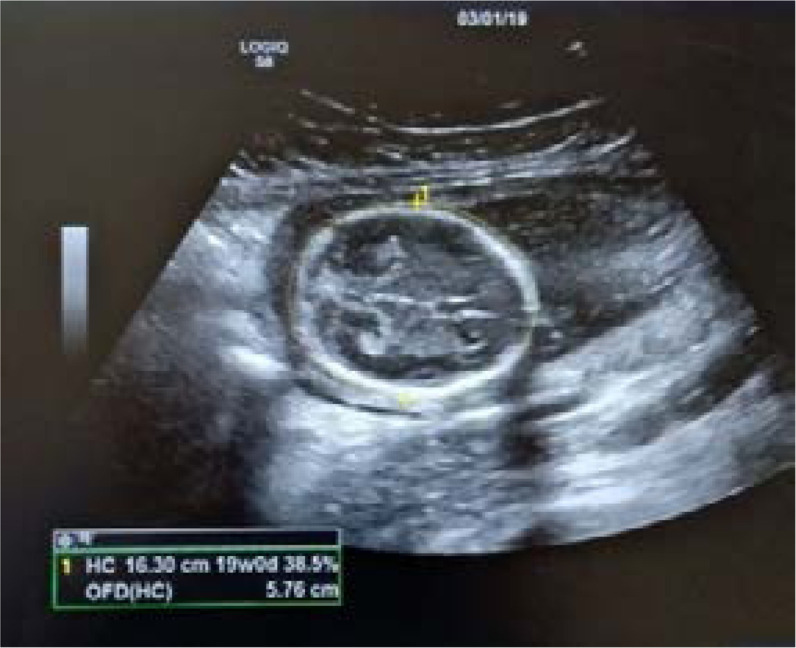
Showing measurement of HC in USG machine.

**HC:** Measurement was taken in the same plane as that of BPD. Measured by tracing along the outer edge of cranium using ellipse method (Fig. 4).

**Fig. 5:**
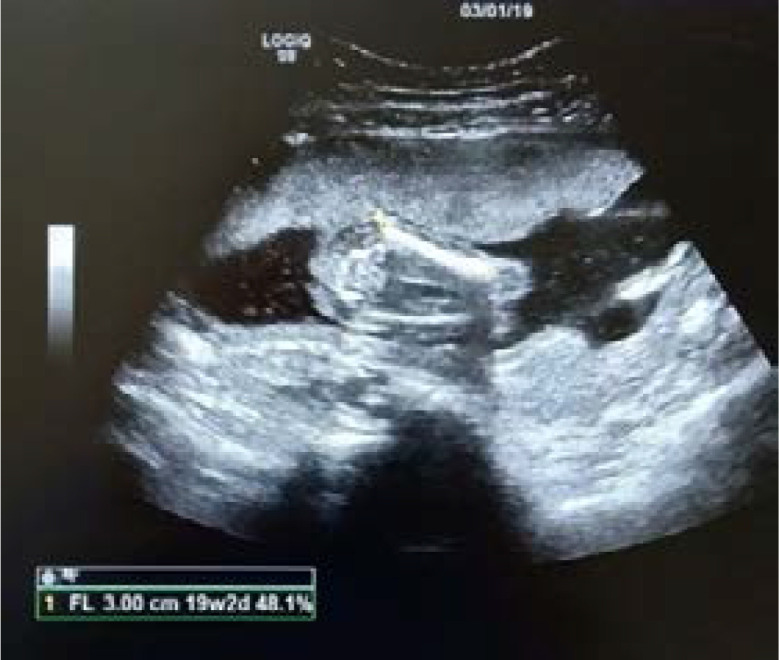
Showing measurement of FL in USG machine.

**FL:** Measurement was taken from the greater trochanter to the lateral condyle (Fig. 5).

## Statistical analysis

The statistical software namely SPSS version 17 statistical package for windows was used for the analysis of data. Microsoft word and Excel have been used to generate graphs, tables etc. In order to ensure that there was no statistically significant difference between the three study groups i.e. 14–20 weeks, 21–30 weeks & 31–40 weeks with regards to age, ‘student t’ test was used. P values less than 0.05 were considered significant. Regression analysis was used to compare correlation of each ultrasonographically measured parameter i.e. TCD, BPD, HC, AC and FL with the gestational age of foetus in normal pregnancy. A reference chart for TCD measurements to its corresponding gestational age developed.

## Results

**Fig. 6:**
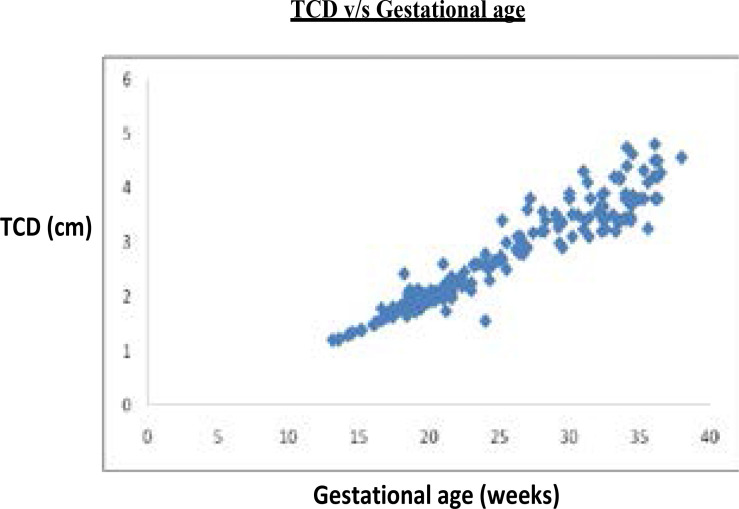
Scattered diagram for TCD to its corresponding GA, showing linear graph indicating stronger correlation.

Three groups of normal pregnant women were made depending upon gestational age of their fetuses i.e. 14–20 weeks, 21–30 weeks and 31–40 weeks. Mean maternal age in all these three groups were 24.8, 24.97 and 25.89 respectively. The ‘p’ value was found to be 0.83 which indicates there is no significant difference in the maternal age between the three groups. Analysis showed a statistically significant linear correlation between TCD and gestational age (R2=0.92, p=0.0006). It is observed that median TCD increased exponentially from second trimester onwards (Fig. 6). A statistically significant linear correlation was also demonstrated between TCD and other measured parameters (Table. 1), and it found to be maximum between TCD and HC (R2=0.91, p=0.0002). Normal range (mean) for TCD relative to gestational age obtained and reference chart for TCD in Indian population made (Table 2).

**Table 1:** Table showing correlation of TCD with BPD, HC, AC and FL in normal pregnancies

PARAMETERS COMPARED	R^2^	P value
TCD Vs BPD	0.909	0.0001
TCD Vs HC	0.915	0.0002
TCD Vs AC	0.914	0.0001
TCD Vs FL	0.656	0.0001

## Discussion

On ultrasonography the characteristic image of cerebellum appears as two lobules on either side of midline in the posterior cranial fossa. In this study it was noted that early sonographic visualization of cerebellum occurred as early as 14 weeks. Transverse cerebellar diameter increases linearly as a function of gestational age and could be used to determine gestational age at any stage of pregnancy. The maximum transverse cerebellar diameter obtained in this study is 47.4 mm (Table 2) which is nearer to the maximum TCD of 48.4 mm obtained from Prabhat et al.^11^, in India. This indicates the close relationship between population of the two different studies, which has same racial characteristics. But maximum TCD of 45.5mm obtained from Vinkesteijn et al.^12^, in Netherland is much lower than our study. This difference may be due to racial, nutrition and environmental variations. Saifon et al.^13^ (2006), found out that human cerebellum is resistant to chronic hypoxemia due to brain sparing phenomenon and in the human fetus, cerebellar growth may be least affected by intrauterine growth retardation (IUGR). This finding is similar to the findings of Jose et al.^14^, which showed that TCD is easier parameter to obtain gestational age of the fetus in certain circumstances such as breech presentation and dolichocephaly (except in anencephaly) where other fetal parameters cannot be used. This implies that transverse cerebellar diameter can assist clinically in the prediction of gestational age of patients who are not sure of last menstrual period (LMP) date. Mcleary et al.^15^, studied the measurement of trans-cerebellar diameter with ultrasonography in 225 normal fetuses ranging from 15 to 39 weeks of gestational age and found it to closely correlated with BPD. They proposed that the trans-cerebellar diameter may be useful in estimating fetal age, particularly in breech presentation where extrinsic pressure may deform the skull and decrease the biparietal diameter. Similar results were found in the current study. There was good correlation between BPD and TCD (R2 = 0.909, p value = 0.0001). Therefore, TCD may be preferred over BPD in assessing gestational age of fetuses in circumstances where head is deformed for e.g. as in molding or dolicocephaly. Lerner J et al.^16^, studied nineteen pregnant women with a clinical suspicion of intrauterine growth retardation and with gestational age confirmed by early ultrasound examination. They found that the transverse cerebellar diameter being consistently correlated with gestational age as predicted by the last menstrual period, whereas most of the other measurements were consistently discrepant with the transverse cerebellar diameter by more than 2.5 weeks (i.e., more than 2 SD above the mean). They therefore concluded that growth of the transverse cerebellar diameter is unaffected by intrauterine growth retardation.

One of the limitation of this study is that sonographic measurement of the TCD in some very active fetuses was difficult and this limited the sample size. Vaginal scan would have decreased the limitation of active baby, but it was not used, because the field of view decreases as the pregnancy advances in age (second and third trimesters). Assessment of TCD in this study was carried out on normal fetuses only; therefore, the effect of IUGR or fetal anomalies on TCD was not assessed. There is scope for more studies in the future to confirm effect of IUGR and fetal anomalies on TCD.

It can be concluded that TCD can be an independent parameter to assess gestational age in fetuses where the other parameters are difficult to be measured. Countries like us where healthcare facilities are limited, need to have their own reference chart for transverse cerebellar diameter. This will prevent misdiagnosis of small or large for gestational age and will avoid unnecessary obstetric intervention during perinatal period.
